# The role of ubiquitination and deubiquitination in the pathogenesis of non-alcoholic fatty liver disease

**DOI:** 10.3389/fimmu.2025.1535362

**Published:** 2025-04-11

**Authors:** Lihui Zhang, Sutong Liu, Qing Zhao, Xiaoyan Liu, Qiang Zhang, Minghao Liu, Wenxiao Zhao

**Affiliations:** ^1^ The First Affiliated Hospital of Henan University of Chinese Medicine, Zhengzhou, China; ^2^ Collaborative Innovation Center of Prevention and Treatment of Major Diseases by Chinese and Western Medicine, Zhengzhou, Henan, China; ^3^ Collaborative Innovation Center of Research and Development on the Whole Industry Chain of Yu-Yao, Zhengzhou, Henan, China

**Keywords:** ubiquitination, deubiquitination, non-alcoholic fatty liver disease, pathogenesis, research progress

## Abstract

Nonalcoholic fatty liver disease (NAFLD) is one of the most common chronic liver diseases and is closely associated with metabolic abnormalities. The causes of NAFLD are exceedingly complicated, and it is known that a variety of signaling pathways, endoplasmic reticulum stress, and mitochondrial dysfunction play a role in the pathogenesis of NAFLD. Recent studies have shown that ubiquitination and deubiquitination are involved in the regulation of the NAFLD pathophysiology. Protein ubiquitination is a dynamic and diverse post-translational alteration that affects various cellular biological processes. Numerous disorders, including NAFLD, exhibit imbalances in ubiquitination and deubiquitination. To highlight the significance of this post-translational modification in the pathogenesis of NAFLD and to aid in the development of new therapeutic approaches for the disease, we will discuss the role of enzymes involved in the processes of ubiquitination and deubiquitination, specifically E3 ubiquitin ligases and deubiquitinating enzymes that are important in the regulation of NAFLD.

## 1 Introduction

Nonalcoholic fatty liver disease (NAFLD) is a pathological syndrome characterized by hepatic metabolic stress injury induced by lipid accumulation and hepatic cell steatosis, excluding alcohol and other clear liver damage factors (1). This usually leads to hepatocellular damage and fibrosis, resulting in non-alcoholic steatohepatitis (NASH) (2). NASH continues to progress towards cirrhosis and hepatocellular carcinoma (HCC) (3). NAFLD is a serious threat to human health and is becoming a more common health issue. It is also linked to the development of certain cardiovascular and cerebrovascular diseases, and is a major cause of end-stage liver disease and liver transplantation (4). According to a recent comprehensive study, the prevalence of NAFLD has increased globally from 25.26% in 1990-2006 to 38.00% in 2016-2019, according to a recent comprehensive study (5). A variety of conditions may combine to lead to NAFLD, which has incredibly complicated causal elements. Different experts and scientists have put out various ideas of pathogenesis in this regard, such as the “multiple strikes” concept and the “second strike” notion (6). Insulin resistance (IR), lipid metabolism imbalance, inflammatory pathway activation, endoplasmic reticulum stress (ERS), mitochondrial dysfunction, lipid metabolism, lipid autophagy, and intestinal microecological imbalance are all involved in the pathogenesis of NAFLD (7). Numerous studies have shown that modifications in protein ubiquitination and deubiquitination are involved in the occurrence and development of NAFLD by affecting IR, ERS, mitochondrial dysfunction, and lipid autophagy. As NAFLD is still becoming more common worldwide, there is an immediate need for integrated strategies to increase awareness and coping strategies for NAFLD at the local, regional, and global levels. To serve as a guide for future studies on medications that target ubiquitination and deubiquitination for the treatment of NAFLD, we present a review of the most recent research on E3 ubiquitin ligases (E3s) and deubiquitinating enzymes (DUBs) and their mechanism of action in NAFLD.

## 2 Ubiquitination and deubiquitination

Ubiquitin (Ub) is a highly conserved polypeptide chain of 76 amino acids that is widely distributed in all cell types (8). Ub has seven lysine residues (K6, K11, K27, K29, K33, K48, and K63), each of which can be ubiquitinated to form a unique form of a multimeric ubiquitin chain (9). In addition, the N-terminal methionine of ubiquitin can also act as a ubiquitination site to form a linear ubiquitin chain. The process of covalent binding of ubiquitin to its substrate proteins is called ubiquitination. Ubiquitination is a multistage enzymatic reaction mediated mainly by the ubiquitin-activating enzyme (E1), ubiquitin-conjugating enzyme (E2), and ubiquitin-protein ligase (E3) and is also a post-translational modification (10). E3 ubiquitin ligases play an extremely important role in determining the timing and specificity of substrate ubiquitination. More than 600 different types of E3 ubiquitin ligases have been identified since the first E3 ubiquitin ligase was discovered by Hershko et al. (11). They can be categorized into three main groups based on their homology with the binding sequences of ubiquitin-conjugating enzymes: RING ubiquitin ligases, HECT ubiquitin ligases, and RBR ubiquitin ligases (12). RING ubiquitin ligases are the most studied ubiquitin ligases and can be divided into two major groups: one with single subunits and the other with multiple subunits (13). HECT ubiquitin ligases can be divided into three subfamilies: NEDD4/NEDD4-like ubiquitin ligases containing the WW structural domain; ubiquitin ligases containing the RLD structural domain (HECT domain and RCC1-like domain- containing protein, HERC), and other E3 ubiquitin ligases that do not contain WW nor RLD structural domains (14). RBR ubiquitin ligases contain an RBR (RING-between-RING) structural domain, which in turn contains two RING-shaped structural domains (RING1 and RING2), with the two RINGs linked by an in-between-RING (IBR) structural domain (15).

DUBs, members of the cysteine protease family, can reverse the process of ubiquitination. They accomplish this by eliminating ubiquitin from substrate proteins. At the N-terminus, proteins have a high degree of selectivity when cleaving heteropeptide links (ubiquitin to lysine) or peptide bonds (ubiquitin to methionine). It is possible to express ubiquitin as a fusion protein that binds to ribosomal subunits or in a multicopy-linked form known as polyubiquitin. These fusion proteins are cleaved by DUBs, resulting in the production of reusable active ubiquitin molecules. Deubiquitination results in the acquisition of new protein functions or changes in protein localization within the cell. Approximately 100 DUBs in the human genome are predicted to possess deubiquitinating enzyme activity. Recent studies tend to categorize DUB into seven families: the USP family, the UCH family, the OUT family, the MJD family, the JAMM family, the ZUP1 family, and the MINDY family (16). The USP family, which has the largest number of members and the most structurally diverse group of known deubiquitinating enzymes, belongs to the cysteine protease class (17). These enzyme molecules contain two short, highly conserved sequences, that is, Cys and His structural domains. It has catalytic triplet residues in its sequence that remove ubiquitin molecules from large protein molecules, eliminate the biological function of ubiquitinated proteins, and play a key regulatory role in DNA damage repair and tumor development (18). Ubiquitination and deubiquitination processes regulate almost all life activities in plants and animals, including cell proliferation, differentiation, apoptosis, DNA replication and repair, transcription, and protein quality control, and are involved in processes such as invasion of pathogens, pathogenicity, and immune response of the human organism (19, 20).

## 3 Ubiquitination and deubiquitination modifications in NAFLD-associated signaling pathways

### 3.1 TAK1 signaling pathway

**Figure 1:**
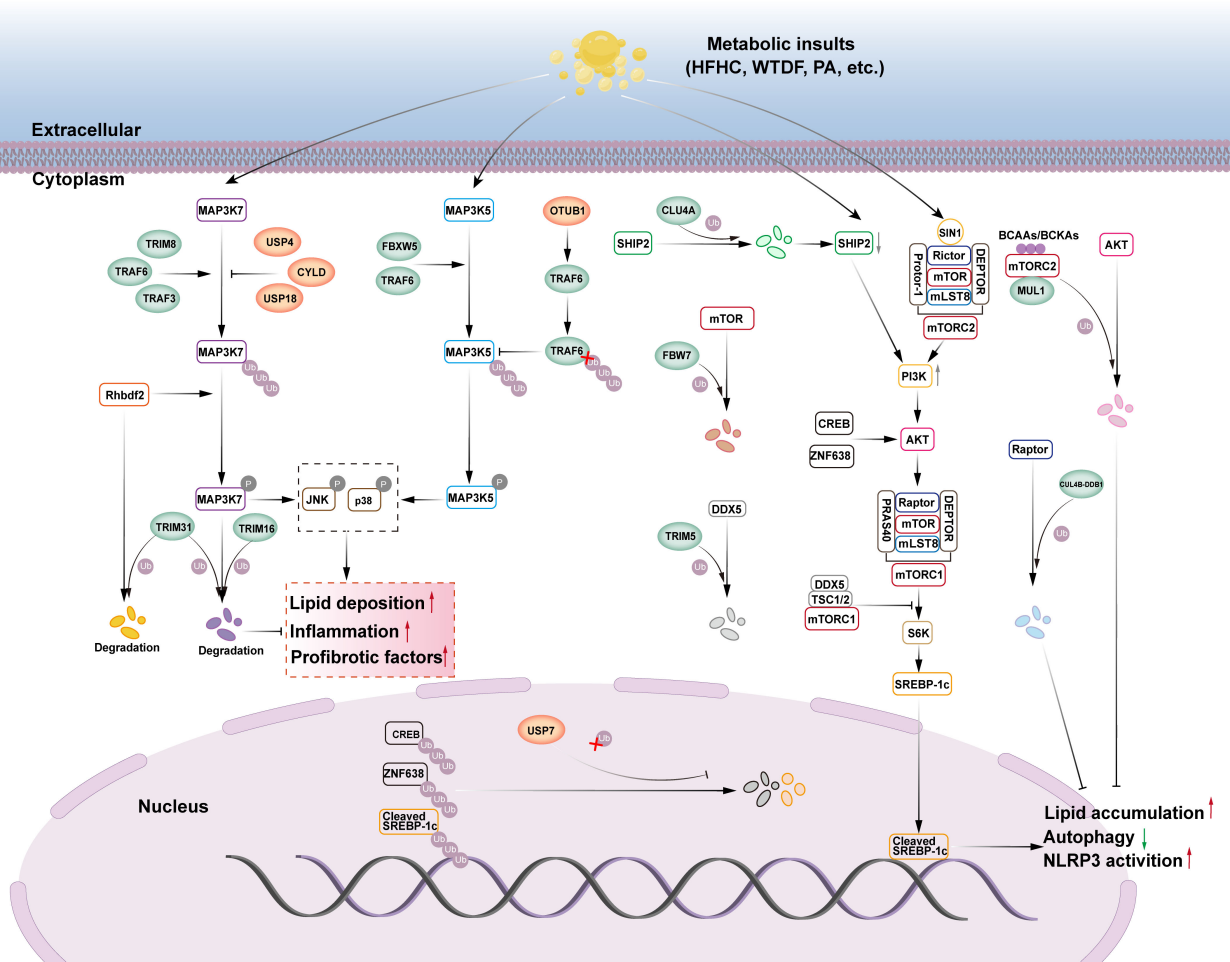
The role of E3s and DUBs in TAK1, ASK1 and mTOR signaling pathway in NAFLD. (1) TAK1, also known as MAP3K7, MAP3K7 promotes lipid deposition, inflammation, and pro-fibrotic factors by phosphorylating its downstream JNK/p38 signaling. The E3 ubiquitin ligases TRIM8, TRAF6, and TRAF3 can ubiquitinate and phosphorylate MAP3K7, activating the downstream JNK/p38 signaling cascade and aggravating liver steatosis, inflammation, and fibrosis. However, the deubiquitinases USP4, USP18, and CYLD can stop this process. Rhbdf2 can activate the MAP3K7 signaling pathway, but TRIM31 can block it through ubiquitination function, which degrades Rhbdf2 and phosphorylates MAP3K7. TRIM16 can also enhance the ubiquitination and degradation of phosphorylated MAP3K7 while inhibiting the downstream JNK/p38 signaling cascade. (2) ASK1, also known as MAP3K5, MAP3K5 promotes lipid deposition, inflammation, and pro-fibrotic factors by phosphorylating its downstream JNK/p38 signaling. The E3 ubiquitin ligases FBXW5 and TRAF6 can ubiquitinate and phosphorylate MAP3K5, activating the downstream JNK/p38 signaling cascade and aggravating liver steatosis, inflammation, and fibrosis. The deubiquitinase OTUB1 directly binds to TRAF6, boosting its deubiquitination, therefore decreasing TRAF6-mediated MAP3K5 ubiquitination and activation, and alleviating NASH. (3) The mTOR signaling pathway can modulate SREBP-1c transcription, inhibit autophagy, promote lipid accumulation, and activate NLRP3. E3 ubiquitin ligase FBW7 can enhance proteasome-mediated degradation of mTOR via ubiquitination, block downstream signal activation, and help avoid NAFLD. CUL4A, an E3 ubiquitin ligase, causes SHIP2 degradation through ubiquitination. The reduction of SHIP2 protein leads to the accumulation of PI3K, which then activates the downstream AKT signaling pathway, promoting adipogenesis, inflammation, and HCC progression. DDX5 may recruit the TSC1/2 complex to mTORC1, preventing downstream signal transduction, whereas the E3 ubiquitin ligase TRIM5 can enhance proteasome-mediated degradation of DDX5 via ubiquitination, reducing its inhibitory effect on mTORC1 signal transduction. MUL1, an E3 ubiquitin ligase, can promote AKT ubiquitination and degradation via BCAAs/BCKAs, inhibiting adipogenesis. CUL4B-DDB1, an E3 ubiquitin ligase, can enhance proteasome-mediated degradation of Raptor via ubiquitination, while inhibiting mTORC1 signaling transduction. USP7 deubiquitinates CREB and ZNF638, triggering the AKT signaling pathway. USP7 can also deubiquitinate Cleaved SREBP-1c, which exacerbates NAFLD.

Transforming growth factor-beta activated kinase 1 (TAK1), also known as mitogen-activated protein kinase 7 (MAP3K7), is an important member of the MAP3K family. TAK1 promotes hepatic steatosis and IR by phosphorylating downstream c-Jun N-terminal kinase (JNK)/p38 and nuclear factor kappa-B (NF-κB) signaling (21). The E3 ubiquitin ligases involved in TAK1 signaling and regulation of NAFLD are TRIM31, TRIM16, TRIM8, TRAF6, and TRAF3, and the DUBs are USP4, USP18, USP13, and CYLD (**Figure 1**).

The TRIM family is a group of proteins with E3 ubiquitin ligase activity that is involved in a variety of intracellular physiological or pathological processes, including cell proliferation, DNA repair, signal transduction, and transcription, by mediating ubiquitination (22). TRIM proteins typically have three structural domains: a zinc finger protein structural domain, one or two B-box structural domains, and a coiled helical structural domain. These structural domains enable TRIM proteins to recognize and tag specific proteins and mediate their degradation, thereby regulating various biological processes within the cell (23). TRIM31 is a key member of the TRIM family and belongs to the single-subunit type of the RING ubiquitin ligases. TRIM31 plays a significant role in the alleviation of inflammation-related diseases (24). The role of TRIM31 in NAFLD has recently been investigated. Surprisingly, hepatically expressed TRIM31 prevented HFD-induced hepatic steatosis, IR, inflammatory response, and associated metabolic syndrome in mice (25, 26). Similarly, in human subjects, the investigators found that TRIM31 tended to be reduced in the livers of patients with NASH compared to human livers without steatosis, and TRIM31 expression was negatively correlated with the severity of NASH (25). The mechanism by which TRIM31 binds with TAK1 and couples the K48-linked ubiquitinated chain to promote TAK1 degradation is as follows: it attenuates hepatic lipid buildup and inflammation by preventing TAK1 activation and its downstream signaling cascade TAK1-JNK1/2-NF-κBp65 in hepatocytes and regulating hepatic metabolic homeostasis, thereby attenuating hepatic lipid accumulation and inflammation (25). According to another study, TRIM31’s CC structural domain aids facilitates its binding to rhomboid family member 2 (Rhbdf2) and encourages Rhbdf2 degradation via K48-linked polyubiquitination. This inhibits Rhbdf2-TAK1 signaling and downstream JNK/NF-κB phosphorylation, and thus exerting a protective effect against NAFLD, including attenuating IRS1 phosphorylation-mediated insulin resistance and thereby inhibiting glucose production. which in turn inhibits glucose production, attenuates hepatic fibrosis by reducing CTGF-mediated deposition of collagen fibers, and attenuates the accumulation of pro-inflammatory factors, cytokines, and chemokines mediated by c-jun phosphorylation, thereby reducing the inflammatory response (26). Another research team discovered a natural compound ‘mulberrin’ in mulberry branches that can effectively target activation of TRIM31, reduce oxidative stress and liver inflammation, thereby alleviating NASH (27). However, instead of using the traditional RING structural domain, TRIM16 uses the B-box structural domain to perform its ubiquitination function. TRIM16 recognizes phosphorylated TAK1 through its B-box structural domains and catalyze K48-linked protein ubiquitination modifications, leading to the degradation of phosphorylated TAK1, which in turn attenuates its phosphorylation of downstream JNK/p38, ameliorates IR, and reduces the expression of genes related to fatty acid metabolism in the liver, and attenuates lipid accumulation and inflammatory response during the progression of NASH (28). This was observed in both a lipotoxicity-induced hepatocyte model and a NASH mouse model. Additionally, TRIM8 participates in several pathogenic mechanisms via the ubiquitination pathway. TRIM8 appears to be a powerful facilitator of NASH progression. Hepatocyte-specific TRIM8 overexpression causes IR, hepatic steatosis, inflammation, and fibrosis, all of which dramatically deteriorate. In contrast, deletion or down-regulation of TRIM8 in hepatocytes led to a significant attenuation of insulin-resistant symptoms in HFD mice, and TRIM8 appeared to be a potent enhancer in the development of NASH (29). The researchers found that TRIM8 was progressively elevated in liver tissue from patients without hepatic steatosis, patients with hepatic steatosis, and patients with NASH (30). Experimental studies have shown that TRIM8-mediated ubiquitination controls the phosphorylation of TAK1 and the downstream JNK/p38 signaling pathway. Overexpression of TRIM8 results in a significant increase in TAK1 phosphorylation, which in turn causes IR and metabolic disorders in HFD-fed NASH mice (30). This implies that inhibition of the ubiquitination pathway of TRIM8 is promising for the treatment of NASH-related metabolic disorders.

TNF receptor-associated factor 6 (TRAF6) is one of the most extensively researched signal transducing members of the TNF receptor-associated factor (TRAF) family of proteins. Several TRAF family proteins are E3 ubiquitin ligases. TRAF6’s ring finger domain (RF) and zinc finger motif (ZF) are crucial for its ubiquitination activity. Currently, it is generally acknowledged that one of TRAF6’s key functions *in vivo* is to catalyze the creation of polyubiquitin chains attached to both TAK1 and itself. This causes TAK1 to become activated, which in turn facilitates the onset of immunological, inflammatory, and cancerous conditions (31). TRAF6 can activate TAK1 by boosting TAK1’s ubiquitination, which exacerbates hepatic steatosis and inflammation. This phenomenon was also confirmed in NAFLD, where researchers used PA to stimulate hepatocytes and found that TRAF6 promotes TAK1 autophosphorylation by catalyzing the K63-linked ubiquitination chain, promoting the expression of adipogenic genes (FASN, SCD1, PPARγ, ACC), inflammatory factors (IL-1β), and chemokine ligand 2 (CCl 2), and thus exacerbating hepatic steatosis and inflammation. This ubiquitination can be inhibited by TIPE2 (32). TRAF3 is a RING ubiquitin ligase that interacts with TAK1 to induce ubiquitination and autophosphorylation of TAK1, which in turn enhances the downstream activation of the inhibitor of nuclear factor kappa-B kinase beta (IKKβ)-NF-κB and MAPK kinase (MKK)-JNK- Insulin receptor substrate 1(IRS1) signaling cascades. TRAF3 belongs to the RING ubiquitin ligases, and TRAF3 mRNA and protein expression in liver samples from NAFLD patients was higher than that of healthy controls. Mechanistically, TRAF3 regulates TAK1 autophosphorylation through a K63-linked ubiquitination chain, which enhances the activation of the downstream IKKβ-NF-κB and MKK-JNK-IRS1 signaling cascades, and simultaneously disrupts the AKT-GSK3β/FOXO1 signaling, exacerbating NAFLD insulin resistance and thus promoting hepatic steatosis and inflammatory responses (33).

The USP family includes USP4, the first deubiquitinating enzyme found in mammalian cells. Numerous signaling pathways that control human pathophysiological processes involve USP4. Interestingly, USP4 has also been linked to NAFLD.NAFLD, and associated metabolic problems may be significantly inhibited by USP4 (34). The protein expression of USP4 in liver tissue samples from patients with NAFLD was significantly lower than that of healthy controls, and mechanistically, USP4 inhibits TAK1 degradation by removing the K48-linked ubiquitinated chain, leading to inhibition of signaling activation of the downstream NF-κB and JNK cascades, which in turn reverses the disruption of IRS-AKT-GSK3β signaling, inhibits IR, and thus attenuates hepatic steatosis and inflammation (35). USP18 is a deubiquitinating enzyme that regulates the course of NAFLD. In the livers of NASH patients, high-fat diet (HFD)-induced obese mice, and genetically obese mice, USP18 expression is down-regulated. Mechanistically, USP18 inhibits TAK1 activation by removing the K63-linked ubiquitination chain. This, in turn, mitigates IR, hepatic steatosis, and inflammatory responses by inhibiting downstream JNK and NF-κB signaling pathways, ameliorates IR, and consequently attenuates hepatic steatosis and inflammatory responses (36). Gradual decrease in protein expression of deubiquitinating enzyme USP13 in hepatic tissue samples from human subjects without hepatic steatosis, with hepatic steatosis, and NASH (37). In the context of metabolic injury, IRHOM2 expression is elevated in hepatocytes and interacts with USP13, which, through its deubiquitination activity, affects the polyubiquitination state of IRHOM2, leading to hyperactivation of downstream signaling pathways (TAK1-JNK and NF-κB p65), exacerbating insulin resistance and mediating pro-inflammatory factors (TNF-α, IL-1b, IL-6, CCL 2, IL-18) are elevated and anti-inflammatory factors (IL-10) are decreased, thereby promoting inflammatory responses (37). Investigators found in hepatocytes that USP13 inhibits lipid accumulation-associated steatohepatitis and inflammation-triggered NASH by directly binding to inactive rhomboid protein 2 (IRHOM2) and modifying IRHOM2 with K63-type deubiquitination, thereby reducing IRHOM2 stability and abundance (37). Cylindromatosis (CYLD) is a member of the USP family with deubiquitinating enzyme activities. To regulate the function of substrate proteins, which in turn regulate numerous signaling pathways such as NF-κB, JNK, and Wnt, CYLD can interact and recognize the substrate protein, excising the ubiquitin chain attached to the recognized substrate protein via lysine at position 63 (K63) (38). Patients with NAFLD or NASH have significantly downregulated CYLD in their livers, and NASH develops more quickly in mice whose hepatocytes have CYLD deletion (39). According to a mechanistic study, CYLD directly interacts with TAK1 and deubiquitinates it, thereby inhibiting the overactivation of the JNK-p38 signaling pathway, which is crucial for the inflammation and metabolism of hyper-nourished NASH (39).

### 3.2 ASK1 signaling pathway

Another member of the MAP3K family is the recombinant apoptosis signal-regulating kinase 1 (ASK1), popularly referred to as MAP3K5. When exposed to pro-inflammatory cytokines, cellular stress, or ERS responses, ASK1 regulates apoptosis and inflammation by activating downstream MKK4/7 and MKK3/6, respectively. This in turn activates JNK and p38 MAPK and regulates both apoptosis and inflammation through the phosphorylation of various target molecules (40). By regulating lipid and glucose metabolism, the ASK1 signaling pathway, which is aberrantly active in patients with obesity and NASH, may drive the progression of non-alcoholic fatty liver (NAFL) to NASH (41). ASK1 inhibition has been demonstrated in many studies to reduce hepatic fibrosis and the inflammatory response in NASH patients and related animal models (42, 43). ASK1 inhibitors have become a common starting point for the development of NASH medications. The E3 ubiquitin ligases involved in ASK1 signaling and the regulation of NAFLD are FBXW5 and TRAF6 and the deubiquitinating enzyme OTUB1 (**Figure 1**).

As a member of the FBXW subclass of F-box proteins, the F-box and WD repeat domain containing five genes (FBXW5) are responsible for breaking down protein substrates linked to a number of pathological processes, such as tumorigenesis, mitotic progression, and inflammation. Hepatocyte-specific FBXW5 overexpression aggravated diet-induced metabolic disorders in the liver and activated ASK1-associated MAPK signaling in the liver, according to an *in vivo* study; however, hepatocyte-specific FBXW5 deficiency significantly inhibited the progression of these abnormalities (44). Mechanistically, FBXW5 increases Lys63-linked ASK1 ubiquitination, aggravating ASK1-JNK/p38MAPK signaling, upregulation of lipid metabolism genes (CD36, PPARα, MCAD, and PDK4), and increase in proinflammatory factors (IL-6, IL-1b, and TNF), which aggravated NASH inflammation and lipid accumulation (44). In addition, TRAF6 promotes the expression and release of potent pro-inflammatory factors (IL-6, CCL5, CXCL8, CXCL3, CXCL10, and NOS2) and pro-fibrotic factors COL1A1, COL3A1, TGFB1, CTGF, VIM, and TIMP1) by activating the JNK1/2-p38MAPK signaling cascade. These factors act synergistically to exacerbate the hepatic inflammatory response and drive the fibrotic process, thereby promoting the pathological progression of NASH (45). Thus, Lys6-linked polyubiquitination of ASK1 by TRAF6 represents a novel mechanism of ASK1 activation in hepatocytes, and could be a potential target for NASH therapy.

The deubiquitinating enzyme OTU domain-containing ubiquitin aldehyde-binding protein Otubain1 (OTUB1) regulates several signaling pathways related to metabolism. In mice, hepatocyte-specific overexpression of OTUB1 dramatically reduced hepatic steatosis, inflammatory response, and liver fibrosis caused by high-fat and high-fat high-cholesterol (HFHC) diets (46). Researchers found that OTUB1 directly binds to TRAF6 and removes K63-linked polyubiquitination modifications on TRAF6 through its deubiquitinating enzyme activity, an effect that inhibits TRAF6-mediated ubiquitination and activation of ASK1, which in turn down-regulates the expression of lipid metabolism-related genes (CD36, Fabp1, Scd1, PPARG and PPARα) and collagen-related genes (Col1a1, Col3a1, and α-SMA), which ultimately attenuated NASH (46).

### 3.3 mTOR signaling pathway

mTOR is a serine/threonine protein kinase from the PI3K-associated kinase (PIKK) family that serves as a catalytic member of two different protein complexes: mTOR complex 1 (mTORC1) and mTOR complex 2 (mTORC2) (47). mTORC1 contains mTOR, Raptor, PRAS40, mLST8, and DEPTOR, whereas mTORC2 contains mTOR, Rictor, SIN1, Protor-1, mLST8, and DEPTOR. mTOR is a key component of both the protein complexes. mTORC1 stimulates *de novo* lipid synthesis via the sterol response element binding protein (SREBP) transcription factor, which regulates the expression of metabolic genes involved in fatty acid and cholesterol biosynthesis and contributes to NAFLD (48). The most significant activity of mTORC2 is the phosphorylation and activation of Akt, which is a critical component of the insulin/PI3K signaling pathway involved in the development of NAFLD (49). The E3 ubiquitin ligases involved in mTOR signaling and NAFLD regulation are FBW7, CUL4B-DDB1, MUL1, CUL4A, TRIM5, and the deubiquitinating enzyme is USP7 (**Figure 1**).

F-box and WD repeat domain-containing7 (FBW7) belong to the F-box family of proteins and are substrate recognition proteins in the multi-subunit RING ubiquitin ligases that target the degradation of key regulators involved in cellular metabolism in a ubiquitin-dependent manner. FBW7 interacts with mTOR, promotes its ubiquitination and proteasomal degradation, regulates lipid homeostasis, and has therapeutic value in preventing and treating associated disorders (50). Apolipoprotein J (ApoJ) may inhibit the ubiquitination of mTOR by FBW7 in NAFLD, resulting in mTOR activation, suppression of lipophagy and lysosomal function, and the occurrence and progression of NAFLD (51). Cullin 4B-Ring E3 ubiquitin ligase (CUL4B-DDB1) is a multi-subunit RING ubiquitin ligase that promotes ubiquitination of Raptor. mTORC1 activates the transcription of genes related to lipid synthesis in the nucleus, SREBP, and recombinant peroxisome proliferator activated receptor alpha (PPARα) via ribosomal protein S6 kinase beta-1 (S6K1) and ribosomal protein S6 kinase beta-2 (S6K2). This promotes lipid synthesis and inhibits fatty acid oxidation, leading to hepatic lipid deposition and fatty liver formation (52). CUL4B-DDB1 inhibits the activation of the mTORC1 signaling pathway by interacting with Raptor and promoting the polyubiquitination of its K48 linkage, an effect that leads to the down-regulation of the expression of genes related to adipogenesis, oxidation, lipid uptake, and secretion (such as, SREBP1, PPARα, CD36, APOE), which attenuates hepatic steatosis in NAFLD (53). MUL1 is a RING finger family ubiquitin ligase found in the outer membrane of mitochondria. The RING finger family ubiquitin ligase has a total length of 352 amino acids, its C-terminal RNF structural domain has both SUMO and ubiquitin ligase activities towards the cytoplasm, and it can interact with proteins on the mitochondrial outer membrane or in the cytoplasm (54). Branched-chain amino acids (BCAAs) and their products, branched chain α-ketoacids (BCKAs), cause Akt degradation in an mTORC2-dependent manner via proteasome-mediated ubiquitination, and MUL1 is important in promoting BCAA/BCKA-induced Akt ubiquitin-dependent degradation, which increases hepatic gluconeogenesis and inhibits hepatic lipogenesis (55, 56). Another study found that MUL1 could modulate Akt protein levels via K48-specific polyubiquitination, thereby regulating body metabolism (57). Cullin 4A (CUL4A) is a protein of the Cullin family that belongs to the multi-subunit RING ubiquitin ligase CRL. Transcriptome analysis of NAFLD-driven hepatocellular carcinoma patients showed that LINC01468 expression was significantly upregulated; LINC01466 is a long non-coding RNA that promotes the progression of NAFLD to HCC by binding to CUL4A and SHIP2, thereby inducing SHIP2 degradation through ubiquitination. The reduction of SHIP2 protein leads to PI3K accumulation, which recruits AKT to the plasma membrane and activates the AKT/mTOR signaling pathway. This activation upregulates adipogenic enzymes (ACLY, FASN, ACAC, SCD1) and the preadipogenic transcription factor SREBP1, driving adipogenesis and HCC progression (58).

DEAD-box protein 5 (DDX5) is an adenosine triphosphate (ATP)-dependent RNA deconjugating enzyme that plays an important regulatory role in tumorigenesis. DDX5 inhibits the transcription of pro-fibrotic genes in hepatic stellate cells and in turn reduces IR in high-fat diet (HFD)-induced obese mice (59). Mechanistic studies have shown that DDX 5 inhibits downstream signaling by recruiting the TSC1/2 complex to mTORC1 to ameliorate NASH progression, and that TRIM5 exacerbates hepatic steatosis and inflammation in NASH by binding to and mediating the ubiquitination and degradation of DDX 5, inhibits autophagy, promotes NLRP 3 inflammatory vesicle activation, and exacerbates NASH liver steatosis and inflammation (60). Hyperforcinol K (HK), a secondary metabolite derived from Hypericum plants, binds directly to DDX5 and prevents its TRIM 5-mediated ubiquitination and degradation, resulting in a significant reduction of hepatic lipid accumulation and inflammation in a NASH mouse model (60). USP7, named for its position as the seventh member of the ubiquitin-specific protease family, regulates hepatic lipogenesis via glucose metabolism. On the other hand, USP7 was able to stabilize cleaved SREBP-1C and form a USP7-ZNF638-cleaved SREBP-1C complex with ZNF638 in the nucleus, thereby regulating the expression of adipogenesis-related genes (61). It can be seen that USP7 ensures the full activation of SREBP-1C and its target genes ACC, FASN, and SCD through a variety of regulatory mechanisms, leading to an increase in hepatic *de novo* lipogenesis and promoting the progression of NASH to cirrhosis and HCC.

### 3.4 SIRT signaling pathway

Silent information regulatory protein (Sirtuin) is an evolutionarily conserved class of proteins that regulates key physiological processes such as apoptosis, metabolism, energy homeostasis, mitochondrial function, and lifespan, and is mainly characterized by nicotinamide adenine dinucleotide (NAD+), dinucleotide (NAD+)-dependent histone deacetylase activity, and mono-ADP-ribosyltransferase activity (61, 62). Scientists have identified seven SIR homologues in mammals, SIRT1–SIRT7 or Sirtuins, which are located in different cellular regions.SIRT1 and SIRT2 are localized in the nucleus and cytoplasm; SIRT3, SIRT4, and SIRT5 are localized in the mitochondrion, whereas SIRT6 and SIRT7 are localized in the nucleus (62). Currently, SIRT1 is the most widely and fully studied protein. SIRT1 attenuates NAFLD through mechanisms such as re-establishment of autophagy, enhancement of mitochondrial function, inhibition of oxidative stress, coordination of lipid metabolism, reduction of hepatocellular apoptosis, and inflammation (63). The biological functions of SIRT6 have been gradually revealed in recent years. SIRT6 regulates lipid metabolism, IR, oxidative stress, and inflammation, making it a possible therapeutic target for metabolic syndrome (64). NASH mice have significantly lower levels of SIRT6 in their livers than normal mice, and the absence of SIRT6 in the liver accelerates hepatic steatosis, IR, and inflammation as well as exacerbates NASH-induced hepatic injury, whereas SIRT6 transgenic mice are unaffected by diet-induced NASH (65).

The gene related anergy in lymphocyte (GRAIL), also known as RNF128, is a RING ubiquitin ligase. GRAIL interacts with SRIT1 and ubiquitinates it, resulting in increased fatty acid absorption and synthesis, and decreased fatty acid β-oxidation. This leads to fatty acid buildup and hepatic steatosis, ultimately leading to NAFLD (66). The anti-NAFLD role of the deubiquitinating enzyme USP22 through stabilization of SIRT1 has also been recently reported (67). Activation of the NKAα1/SIRT1/autophagy signaling pathway can alleviate NAFLD by regulating fatty acid oxidation and lipogenesis (67). In a high-fat diet-induced NAFLD mouse model, researchers found that instability of the membrane protein NKAα1 and the resulting NKA dysfunction led to proteasome-mediated degradation of SIRT1 via ubiquitination, blockade of the autophagy signaling pathway, and exacerbation of hepatic steatosis, a process that could be inhibited by the deubiquitylating enzyme USP22 (68). Enhanced expression of the deubiquitylating enzyme USP22 leads to SIRT1 deubiquitination and maintains its stability, which in turn activates the downstream autophagy signaling pathway, exerting a function in boosting fatty acid oxidation and inhibiting adipogenesis for the treatment of NAFLD (67, 68). Adipose triglyceride lipase (ATGL) is a key hepatic lipase that initiates triglyceride (TG) catalysis in rodents and human cells (69). ATGL overexpression in the liver promotes fatty acid (FA) oxidation and ameliorates hepatic steatosis (70). ATGL regulates hepatic lipid droplet catalysis and FA oxidation primarily by activating autophagy/lipophagy via SIRT1 (71). COP1, also known as RFWD2, is a substrate receptor molecule of the multi-subunit cullin-RING ubiquitin ligase CRL4COP1/DET1 complex. The E3 ubiquitin ligase COP1 was found to bind to the consensus VP motif of ATGL and target it for proteasomal degradation via K-48-linked polyubiquitination, reducing its stability and resulting in fatty acid accumulation and hepatocyte lipid accumulation (72).

USP10 suppresses ubiquitinated protein degradation and regulates cellular metabolism, inflammation, and other processes (73). USP10 has been demonstrated to inhibit palmitate-induced hepatic steatosis in HepG2 cells, and its absence results in a large increase in lipid droplets in cells (74). SIRT6 expression was lower in liver samples from patients with NAFLD than in normal human liver tissues (75). SIRT6 knockdown in NASH mouse models led to increased transcription and expression of NF-κB target genes as well as higher levels of inflammatory factors such as TNF-α and IL-1β in the liver. Conversely, overexpression of SIRT6 reduces inflammatory factor expression in the livers of mice (76, 77). A previous study revealed that SIRT6 inhibits SREBP1 and SREBP2 via at least three pathways (65). SREBP is a transcription factor that regulates lipid homeostasis in hepatocytes, and its overactivation promotes NAFLD incidence and progression. SIRT6 is reduced by proteasome-mediated degradation via ubiquitination in the hepatocyte cytoplasm when NAFL progresses to NASH in NAFLD patients compared to healthy individuals (65, 75). Clinical studies have found that USP10 is lowly expressed in liver tissue samples from NAFLD patients, and further studies have shown that USP10 inhibits proteasome-mediated degradation of SIRT6 via deubiquitination. This inhibition leads to downregulation of mRNA levels of SIRT6 target genes ACC1, SCD1, FAS, and CRFVL6, and reduced serum inflammatory factors TNF-α, IL-1β, IL-4, IL-6, IL-2, and chemokine MCP-1, which in turn attenuates hepatic steatosis and inflammation in HFD mice (78). It can be seen that targeting to increase the expression of USP10 stabilizes SIRT6, increases its activity, inhibits SREBP1 and SREBP2 activity, reduces lipid synthesis and accumulation, and thus prevents the progression of NAFLD.

### 3.5 TGF-β signaling pathway

**Figure 2:**
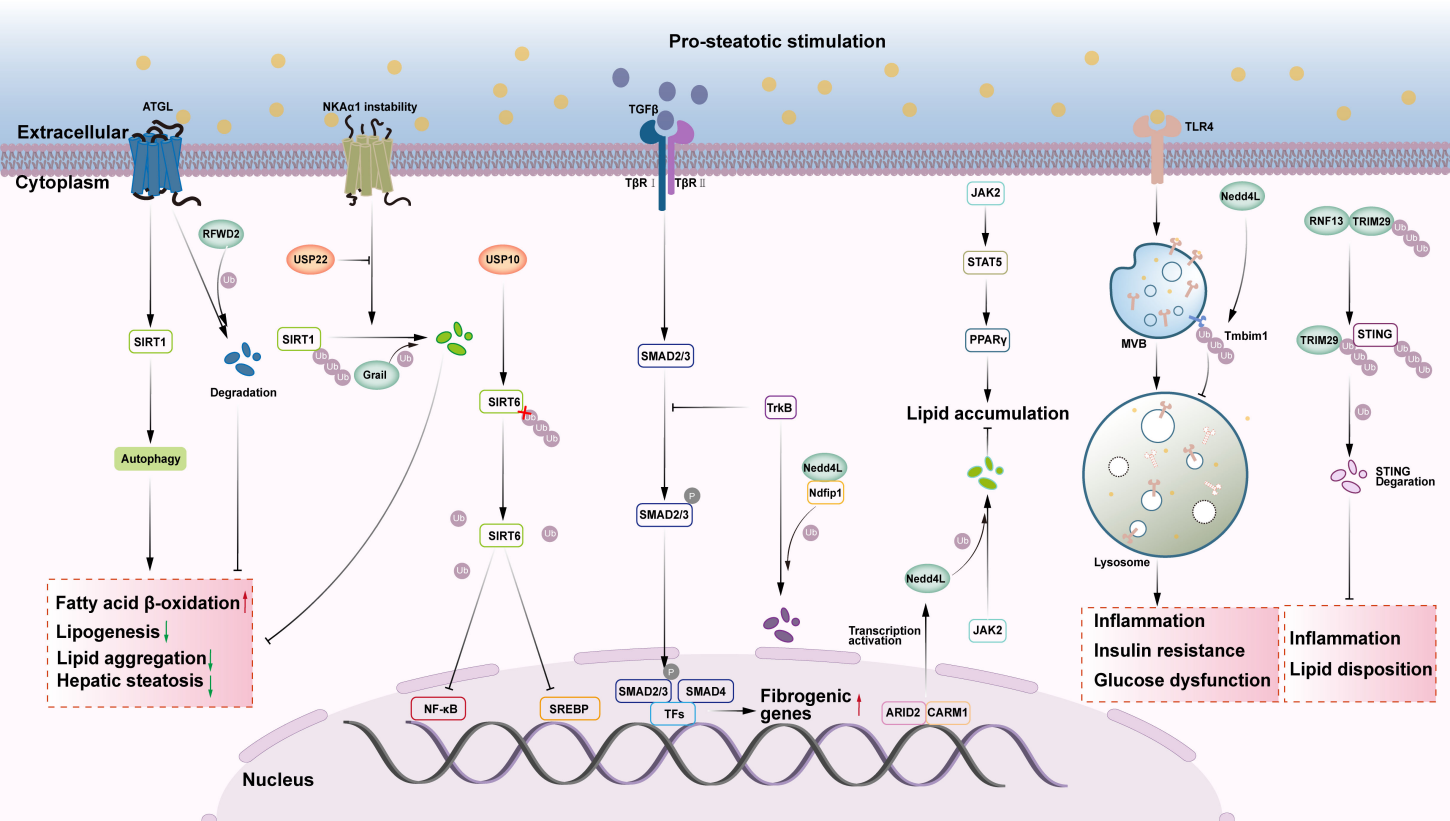
The role of E3s and DUBs in SIRT, TGF-β, JAK, TLR4 and STING signaling pathway in NAFLD. (1) ATGL stimulates the autophagy signaling pathway via SIRT1, promotes fatty acid beta oxidation, and decreases liver steatosis. E3 ubiquitin ligase RFWD2 targets ATGL proteasome degradation, while E3 ubiquitin ligase Grail targets SIRT1 proteasome degradation, which increases FA and hepatic lipid accumulation. The deubiquitinase USP22 can deubiquitinate SIRT1, stabilize SIRT1, and have anti-NAFLD actions. USP10, a deubiquitinase, can prevent NAFLD by stabilizing SIRT6 and blocking downstream signaling pathways such as NF-κB and SREBP. (2) TrkB can suppress the TGF-β/Smad signaling pathway, alleviating NASH-related liver fibrosis. Ndfip1 can ubiquitinate and degrade TrkB via the E3 ubiquitin ligase Nedd4L, aggravating NASH-associated liver fibrosis. (3) The activation of JAK2/STAT5/PPAR gamma axis increases hepatic lipid accumulation. ARID2 can impede downstream signal transduction by ubiquitinating and degrading JAK2 via the E3 ubiquitin ligase Nedd4L. (4) TLR4 can activate the MVB lysosome signaling pathway, causing inflammation, insulin resistance, and glucose metabolism disorders, aggravating NASH. The E3 ubiquitin ligase Nedd4L activates Tmbim1 via ubiquitination, boosting lysosomal degradation of TLR4 and improving NASH. (5) The E3 ubiquitin ligase RNF13 catalyzes TRIM29, increasing its stability. Ubiquitinated TRIM29 causes STING to be degraded by proteasomes, which inhibits hepatic lipid accumulation and inflammation.

Transforming growth factor-β (TGF-β) is a member of the TGF-β superfamily that regulates cell growth and differentiation. It is also a key factor in liver fibrosis (79). It also plays a significant role in the progression of NAFLD-related liver fibrosis. Overexpression of TGF-β in NAFLD or NASH activates the Smad signaling pathway, leading to apoptosis, hepatic stellate cells (HSCs) activation and proliferation, and myofibroblast (MFB) transformation, resulting in increased extracellular matrix (ECM) deposition (80). Smad proteins, the only known downstream cytosolic transnuclear substrates for TGF-β, have numerous isoforms, including Smad2 and Smad3, which act as receptor regulators with prohepatic fibrogenic effects (81). Nedd4L is the only E3 ubiquitin ligase that regulates the progression of hepatic fibrosis in NAFLD by modulating key components of the TGF-β signaling pathway (**Figure 2**). Nedd4L, also known as Nedd4-2, is an E3 ubiquitin ligase of the Nedd4 family, which belongs to the HECT subtype. Tyrosine kinase receptor B (TrkB) protein expression in HSCs diminishes as fibrosis progresses and is a key inhibitor of NASH-related liver fibrosis (79). TrkB inhibits TGF-β/Smad signaling, thereby slowing the progression of NASH-related liver fibrosis. The TGF-β/Smad signaling pathway mediates the ubiquitinated degradation of TrkB proteins through the E3 ubiquitin ligase Nedd4-2 (79). Previous research has demonstrated that Ndfip1 increases TrkB ubiquitination by recruiting Nedd4-2 (82). This study also confirmed that Ndfip1 is a ‘bridge’ between TrkB and Nedd4-2. The proteasome-mediated degradation of TrkB via ubiquitination activates the TGF-β/SMAD signaling pathway, which up-regulates the expression of α-SMA, COL1A1, TIMP1, TGFβ1, and CTGF genes, and promotes the accumulation of ECM (79). TGF-β-driven Ndfip1 expression increases TrkB ubiquitination through Nedd4-2 in HSCs, thereby aggravating the progression of NASH-associated hepatic fibrosis. These findings not only reveal the important role of TrkB in NASH-associated liver fibrosis, but also provide a theoretical basis for the development of new therapeutic targets. However, although these studies provide important clues to the role of TrkB in liver fibrosis, there is still a lack of data from clinical studies targeting TrkB, and its differential expression and therapeutic potential in human NASH patients still need to be further validated.

### 3.6 JAK signaling pathway

The JAK-STAT pathway regulates inflammation and tumorigenesis. This signaling pathway affects lipid metabolism in adipose tissue by influencing PPARγ expression and worsening hepatic steatosis (83, 84). This contradicts previous findings on PPARγ (85). This could be due to the signal being activated by various factors and their interactions with other signaling pathways. Recent investigations have shown that stimulation of the JAK2-STAT5-PPARγ axis enhances hepatic lipid accumulation, which can be blocked by AT-rich interaction domain 2 (ARID2)-mediated protein ubiquitination (86) (**Figure 2**). ARID2 recruits coactivator-associated arginine methyltransferase1 (CARM1) to increase H3R17me2a levels at the promoter of E3 ubiquitin ligase Nedd4L. This activated the transcription of Nedd4L, which mediates proteasome-dependent degradation of JAK2 via ubiquitination. This inhibits the downstream STAT5-PPARγ signaling pathway and attenuates HFD-induced hepatic steatosis (86).

### 3.7 Innate immune response

TLR4 signaling pathway: Previous studies have demonstrated that TLR4 affects critical signaling pathways such as inflammation, IR, and metabolic balance, principally through the regulation of IKK/NF-κB and MAPK, which govern NASH (87, 88). Recent research has shown that TLR4 mediates the MVB-lysosomal signaling pathway, which affects inflammation, IR, and impaired glucose metabolism, exacerbating NASH, which can be inhibited by ubiquitination of Tmbim1 by the E3 ubiquitin ligase Nedd4L (89) (**Figure 2**). Tmbim1, a membrane protein found in MVBs and lysosomes, is an inhibitory adipogenic factor, and some studies have shown that it plays an important negative regulatory function in adipogenesis and obesity-related metabolic diseases (90). Researchers have also discovered that the E3 ubiquitin ligase Nedd4L activates Tmbim1 through ubiquitination. Activated Tmbim1 binds to the ESCRT endosomal sorting complex, promoting the formation of multivesicular bodies (MVBs), which facilitates the lysosomal degradation of TLR4. Moreover, the expression of Tmbim1 in hepatocytes significantly inhibits high-fat diet-induced insulin resistance, hepatic steatosis, and inflammation in mice, ultimately ameliorating NASH (89).

STING signaling pathway: The STING signaling pathway has emerged as a key mediator of inflammation in the setting of infection, cellular stress, and tissue injury, and inhibitors targeting the STING pathway have the potential to benefit humans from a number of inflammatory diseases (91). STING signaling is an important intracellular pathway for worsening metabolic dysregulation in patients and mice with NASH and NAFLD (92). Using a phenotype-based high-content imaging analysis system, researchers screened for E3 ubiquitin ligases that inhibit lipid deposition and identified RNF13 as a potential candidate. Subsequent experiments showed that RNF13 improves glycolipid metabolism, inflammatory damage, and fibrosis in NAFLD by inhibiting the natural immune cGAS-STING pathway (93) (**Figure 2**). In the mechanistic study, authors discovered that RNF13 firstly increases the stability of TRIM29 protein by catalyzing K63-type ubiquitination, and then the latter induces the proteasomal pathway degradation of STING by catalyzing K48-type ubiquitination, which inhibits the activation of the cGAS-STING pathway, and blocks the inflammatory signaling pathways such as Toll-like receptor, the lipid metabolism signaling pathway, and the hepatic fibrosis signaling pathway blocking the transduction of inflammatory signaling pathways such as Toll-like receptors, lipid metabolism signaling pathways and hepatic fibrosis signaling pathways to attenuate hepatic lipid accumulation and inflammatory responses (93). This study revealed that natural immune response is a key driver of NAFLD progression. Given that there are few natural immune-related inhibitors or agonists in clinical trials for NAFLD, modulating the natural immune response activated in NAFLD could represent an underexplored and promising area for therapeutic research in NAFLD.

### 3.8 Endoplasmic reticulum stress

The ER is an important continuous-membrane organelle consisting of a series of flat vesicles connected to the nuclear membrane, the quality control of which plays an important role in the maintenance of cellular homeostasis. Endoplasmic reticulum stress (ERS) is induced when cells are exposed to unfavorable external stimuli (conditions such as genetic mutation, hypoxia, nutrient deficiency, and oxidative stress), leading to the accumulation of unfolded and misfolded proteins in the lumen of the ER, which activates the unfolded protein response (UPR) to defend against unfolded proteins (94). Quality control in the ER primarily targets the proteins synthesized within the ER and is mediated by two main pathways: the autophagy-lysosome system and the ubiquitin-proteasome system. The second system is known as the “ER-associated protein degradation (ERAD)” pathway (95).

**Figure 3:**
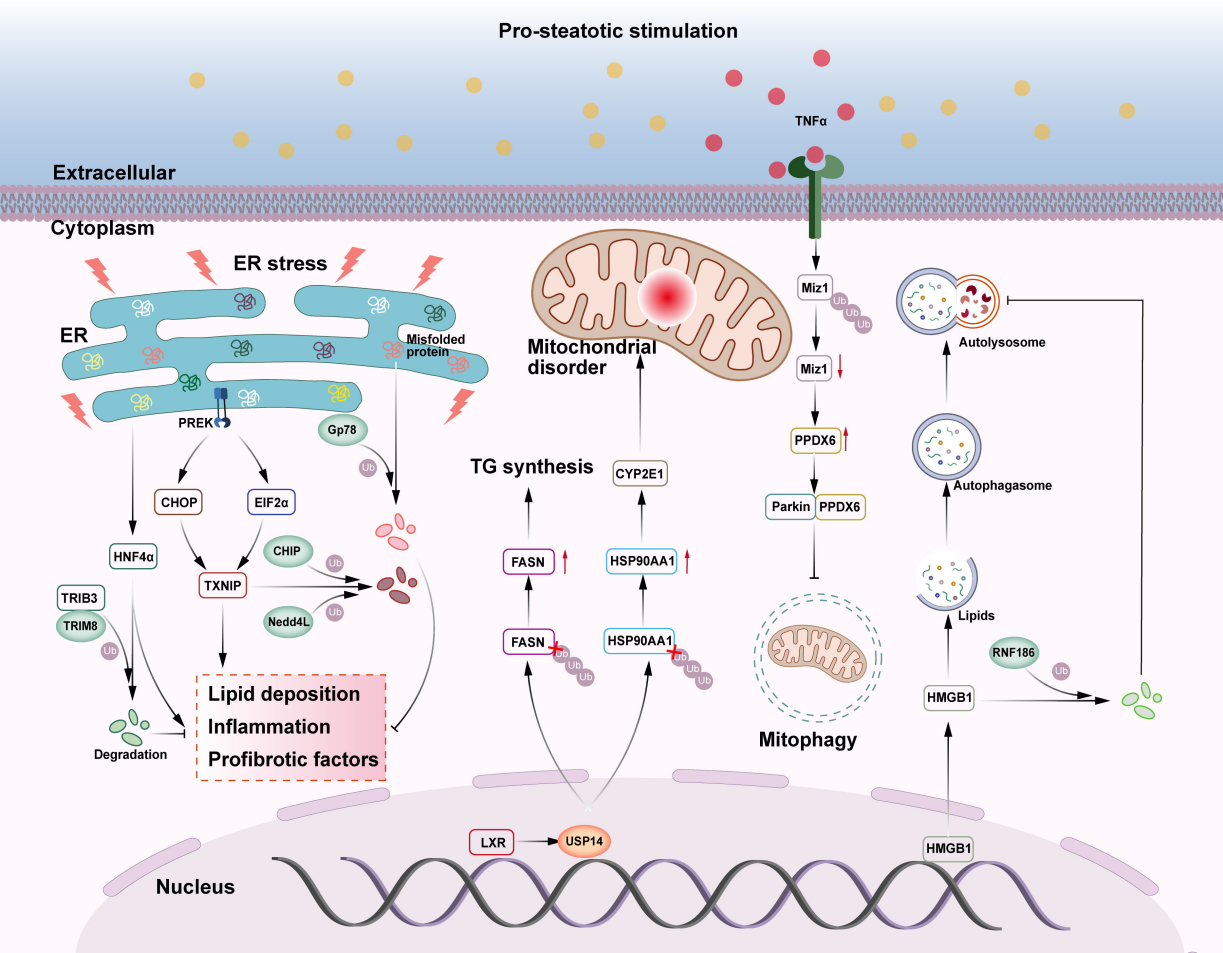
The role of E3s and DUBs in NAFLD-related ERS, mitochondrial malfunction, and lipid autophagy. (1) E3 ubiquitin ligases CHIP and Nedd4L can ubiquitinate and degrade TXNIP in the endoplasmic reticulum stress pathway, inhibiting liver lipid deposition, inflammation, and fibrosis. TRIB3 recruits E3 ubiquitin ligase TRIM8 to degrade HNF4α and prevent NAFLD. E3 ubiquitin ligase Gp78 catalyzes the ubiquitination and degradation of unfolded proteins in the endoplasmic reticulum, relieving endoplasmic reticulum stress and inhibiting NASH and HCC. (2) The deubiquitinase USP14 deubiquitinates both FASN and HSP90AA1, boosting TG production and mitochondrial dysfunction, aggravating liver steatosis, inflammation, and fibrosis. Miz1, which promotes mitochondrial autophagy, can be degraded by ubiquitination, thereby inhibiting mitochondrial autophagy and exacerbating NASH. (3) HMGB1 promotes lipid autophagy, degrades lipid droplets, and helps to avoid NAFLD. In NAFLD, HMGB1 can be ubiquitinated and degraded by E3 ubiquitin ligase RNF186, thereby promoting the development of NAFLD.

CHIP is a U-box family ubiquitin ligase involved in ERS-related ubiquitination and degradation of proteins (96) (**Figure 3**). Increasing evidence suggests that CHIP is involved in metabolic pathways such as liver injury and NASH (97, 98). Thioredoxin-interacting protein (TXNIP) is a key regulator of glucose metabolism disorders, cholesterol accumulation, and fatty acid synthesis in the liver (99), modulating ERS, and leading to hepatic steatosis and inflammation. This pathological process can be ameliorated by CHIP and Nedd4L through ubiquitination of TXNIP, leading to its degradation and the consequent inhibition of ERS (100, 101). Gp78 is a RING ubiquitin ligase that regulates ERAD during ER homeostasis (**Figure 3**). Gp78-/- mice show upregulation of the unfolded protein response (UPR) pathway and SREBP-1 regulation of *de novo* lipogenesis and hepatic tissue steatosis, inflammation, and fibrosis in steatohepatitis, followed by the development of HCC. Further studies revealed that the E3 ligase Gp78 regulates ERAD by ubiquitination of misfolded ER proteins, blocks UPR-driven activation of the lipid synthesis gene SREBP-1, and inhibits the development of NASH and HCC (102). As previously stated, TRIM8 can bind to the substrate TAK1 and regulate its related signaling pathways, worsening the incidence and progression of NASH (30). Recent research has also indicated that TRIM8 plays a role in ER stress-induced NAFLD (**Figure 3**). Tribbles homolog 3 (TRIB3), an ERS and metabolic sensor, is important in chronic inflammation and cancer because it inhibits ubiquitin-mediated substrate degradation (103). TRIB3 can directly interact with hepatocyte nuclear factor 4 alpha (HNF4α) and mediate ER stress-induced degradation of HNF4α. Inhibition of HNF4α degradation attenuates the effect of TRIB3 on high fructose and high fat diets (HFF)-induced NAFLD in mice. In this process, TRIM8 acts as an E3 ubiquitin ligase that promotes the ubiquitination and degradation of HNF4α, thereby enhancing TRIB3-mediated regulation of HNF4α (104). It is evident that TRIM8 is able to be recruited by TRIB3 and catalyzes the polyubiquitination of HNF4α at the K48 linkage on lysine 470, whereas interfering with the TRIM8-TRIB3-HNF4α interaction may regulate lipid metabolism-related genes (FABP3, DGAT2, CYP7A1, CES1, CES2, PPARα, APOB), enhancing fatty acid oxidation, reducing lipid synthesis and accumulation, promoting cholesterol metabolism and bile acid secretion, and ultimately ameliorating hepatic lipid metabolism disorders, which could provide a new potential approach for the treatment of NAFLD or even other liver diseases (104).

### 3.9 Mitochondrial dysfunction

Mitochondrial dysfunction, which includes altered mitochondrial morphology, mitochondrial DNA damage, disruptions in fatty acid and energy metabolism, oxidative stress, lipid peroxidation, and mitochondrial autophagy abnormalities (105), plays a significant role in the pathogenesis of NAFLD. As a result, investigation of liver mitochondria has become a new and significant advancement in the fight against NAFLD.

USP14, an important regulator of the ubiquitin-proteasome and autophagy pathways, not only regulates classical life activities such as cell cycle, immune response, and signaling, but also plays a fine-grained regulatory role on key proteins in mitochondrial oxidative stress-related pathways (106) (**Figure 3**). Previous research has demonstrated that hepatic USP14 expression is much higher in NAFLD patients and that hepatic USP14 overexpression exacerbates diet-induced hepatic steatosis, inflammation, and fibrosis in mice, in contrast to the results of hepatic USP14 knockdown (107, 108). The liver X receptor (LXR) enhances the transcription of USP14, which increases its protein level. USP14 further promotes FASN deubiquitination and inhibits FASN breakdown, boosting TG production and fatty liver (107). In another study, immunoprecipitation and ubiquitination analyses demonstrated that USP14 inhibited the degradation of heat shock protein 90alpha family class A member 1 (HSP90AA1) by decreasing its lysine 48-linked ubiquitination, while upregulation of the HSP90AA1 protein promoted the accumulation of the CYP2E1 protein, and that CYP2E1 accumulation was one of the potential causes of excessive hepatic ROS production (106). These data imply that USP14 is closely linked to hepatic steatosis and oxidative stress.

Mitochondrial autophagy is a type of autophagy regulated by Parkin and PTEN-induced potential kinase protein 1 (PINK1), which is involved in a variety of physiological and pathological processes in the body (109). Mitochondrial dysfunction is linked to decreased mitochondrial autophagy, which exacerbates NASH (110). In NASH livers, the absence of hepatocyte myc-interacting zinc finger protein 1 (Miz1) causes an increase in free recombinant peroxiredoxin 6 (PRDX6) levels. Parkin preferentially binds to PRDX6 and inhibits the PINK1/Parkin mitochondrial autophagy pathway, resulting in the production of pro-inflammatory cytokines by hepatic macrophages, including TNF-α and IL-1β (111). Increased TNF-α production can reduce hepatocyte Miz1 expression by E3 ubiquitination, creating a positive feedback loop that triggers NASH. It remains unclear whether TNF-α-induced Miz1 degradation is directly mediated by Parkin or if other E3 ubiquitin ligases are involved (111).

### 3.10 Lipid autophagy

To regulate abnormalities in lipid storage, the organism undergoes a selective form of autophagy, lipophagy, which specifically targets a unique type of neutral lipid storage organelle, lipid droplets (LDs) (112). Lipophagy can selectively recognize and degrade lipids and plays an important role in regulating cellular lipid metabolism and maintaining intracellular lipid homeostasis. The lipophagy process is subject to the direct or indirect regulatory effects of various factors such as genes, enzymes, and transcriptional regulators (113). The lipid autophagy process in hepatocytes can effectively degrade lipid droplets, thereby reducing lipid accumulation and preventing NAFLD (114).

RNF186 is a RING ubiquitin ligase that induces ER stress in hepatocytes, impairing insulin sensitivity, and inducing hepatic steatosis (115) (**Figure 3**). In addition, hepatocyte-specific RNF186 knockout mice were protected from high-fat diet (HFD)-induced obesity and hepatic inflammatory responses (116). A high-fat diet induced the translocation of high mobility group protein 1 (HMGB1) from the nucleus to the cytoplasm and induced hepatocyte lipid autophagy. In NAFLD, RNF186 expression was consistently upregulated and mediated the degradation of cytoplasmic HMGB1 by ubiquitination of the K48- and K63-chain, which suppressed hepatocyte lipid phagocytosis and contributed to the development of NAFLD (117). This study demonstrates RNF186’s mechanism of action in terms of cytosolic lipophagy and identified a viable therapeutic target for the treatment of NAFLD.

## 4 Other E3 ubiquitin ligases and deubiquitinating enzymes

**Figure 4:**
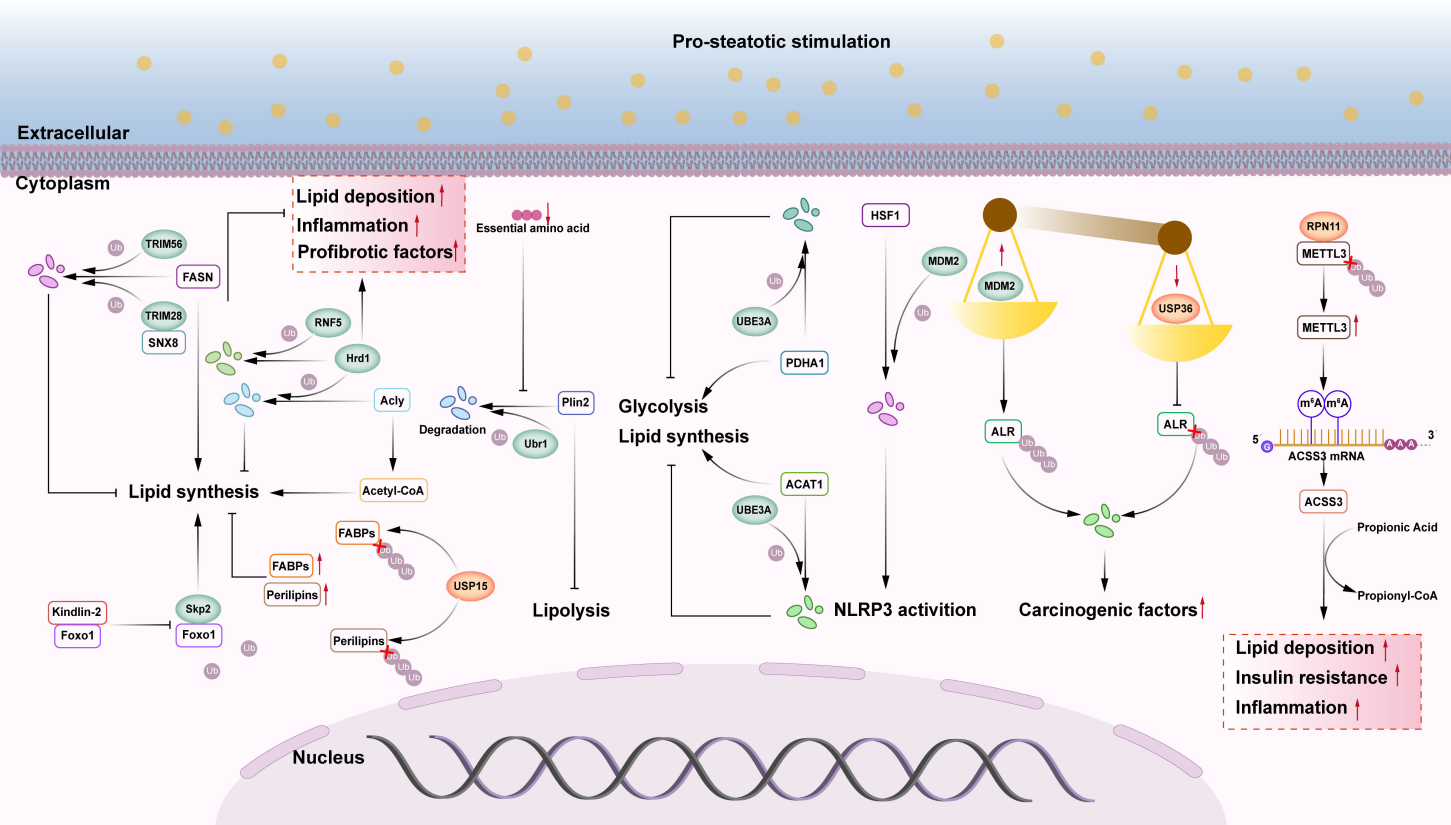
The role of other E3s and DUBs in NAFLD. (1) FASN may activate hepatic lipid synthesis, SNX8 can recruit the E3 ubiquitin ligase TRIM28 to degrade FASN protein, and TRIM56 can also trigger FASN proteasomal pathway degradation, slowing the progression of NAFLD. (2) Kindlin-2 can bind to Foxo1 and inhibit Skp2 mediated ubiquitination of Foxo1 protein, enhancing its protein stability and leading to hepatic steatosis. (3) Acly is an enzyme that facilitates the biosynthesis of fatty acids. Hrd1, an E3 ubiquitin ligase, ubiquitinates and degrades Acly, which inhibits liver fat synthesis. Other research has found that overexpression of Hrd1 worsens hepatic steatosis, inflammation, and fibrosis. The E3 ubiquitin ligase RNF5 can ubiquitinate and degrade Hrd1, slowing the progression of NASH. (4) USP15 deubiquitinates FABPs and Perilipins, increasing their stability and promoting liver lipid accumulation. (5) E3 ubiquitin ligase Ubr1 improves hepatic steatosis by degrading Plin2. When essential amino acids are deficient, Ubr1 becomes inactive, and increased Plin2 protein levels limit liver lipolysis, resulting in NAFLD in lean individuals. (6) The E3 ubiquitin ligase UBE3A can enhance proteasome-mediated degradation of PDHA1 and ACAT1 via ubiquitination, while also inhibiting glycolysis and lipid synthesis. (7) The E3 ubiquitin ligase MDM2 enhances proteasome-mediated degradation of HSF1 via ubiquitination, decreasing its inhibitory impact on NLRP3 and activating NLRP3 inflammasomes. (8) MDM2 promotes proteasome-mediated degradation of ALR via ubiquitination, whereas USP36 deubiquitinates ALR. The imbalance between ubiquitination and deubiquitination of ALR causes severe degradation, and ALR depletion is a major exacerbating factor in the progression of NASH-HCC. (9) The deubiquitinase RPN11 stabilizes METTL3, increases m6A modification and ACSS3 expression, upregulates lipid metabolism genes via histone acetylation to generate acetyl CoA, and promotes liver lipid accumulation, IR, and inflammation.

Ubiquitination and deubiquitination play important roles in the signaling pathways involved in the occurrence and development of NAFLD. In fact, there are some other E3 ubiquitin ligases and deubiquitinases that are still involved in the occurrence and development of NAFLD, although they do not play a role in the NAFLD related signaling pathway (**Figure 4**).

FASN is a critical enzyme in the adipose *de novo* pathway in NASH patients, where it is primarily responsible for synthesizing extra fat and triggering fibrosis and inflammation (118). SNX8 is a key inhibitor of NAFLD, which mediates FASN protein degradation through recruitment of TRIM28 and enhancement of TRIM28-FASN interactions, regulates lipid metabolism genes (FXR, ACACA, HADC 2, FXR VL 5, SCD 1), and inhibits hepatic lipid deposition. SNX8 is a direct interacting and repressor of FASN, a key rate-limiting enzyme in the *de novo* synthesis of fatty acids, which plays an important role in the pathogenesis of NAFLD (119). Therefore, targeting the SNX8-FASN axis to regulate hepatic lipid metabolism is one of the most attractive targets for the prevention and treatment of NAFLD (120). Another study utilized a high-content imaging analysis system to identify the E3 ubiquitin ligase TRIM56 as a suppressor of lipid deposition. The study experimentally demonstrated that TRIM56 promotes the degradation of FASN via the proteasomal pathway by catalyzing K48-linked ubiquitination, thereby suppressing FASN protein expression and downstream lipid synthesis to delay the progression of NAFLD (121). HMG-CoA reductase degradation protein 1 (Hrd1) functions as both an E3 ubiquitin ligase and ERAD complex subunit. ATP citrate lyase (Acly) catalyzes fatty acid production. Hrd1 interacts with and ubiquitinates Acly, reducing its protein levels, inhibiting acetyl coenzyme A levels, and suppressing lipogenesis; in db/db mice and isolated mouse primary hepatocytes, hepatic Hrd1 expression was negatively correlated with NAFLD, whereas Hrd1 overexpression enhanced insulin sensitivity, ameliorated hepatic steatosis (122). However, another study found that Hrd1 overexpression dramatically enhanced hepatic steatosis, the inflammatory response, and fibrosis in diet-induced NASH mice (123). RNF5 is a member of the ubiquitin ligase family and is mainly localized to the ER. A study showed that RNF5 inhibited the progression of NASH by down-regulating lipid metabolism genes (CD36, SCD1, ACACA, and PPARG) and inflammatory genes (IL-6, CXCL2, and TNF) through targeted degradation of Hrd1 via the ubiquitin-mediated proteasome pathway (123). Additional research is required to determine whether Hrd1 plays a positive or negative regulatory role in NAFLD/NASH. Ubiquitin protein ligase E3A (UBE3A), also known as E6AP, is an HECT-like ubiquitin ligase. UBE3A can induce proteasome-mediated degradation of pyruvate dehydrogenase E1 component subunit alpha 1 (PDHA1) via ubiquitination to inhibit glycolysis, while also interacting with acetyl-coenzyme A acetyltransferase 1 (ACAT1), resulting in proteasome-mediated degradation of ACAT1 via ubiquitination. This leads to increased accumulation of triglycerides, cholesterol, and ketone bodies (124). A previous study revealed an important mechanism that regulates the stability and enzymatic activity of PDHA1 and ACAT1 in cells via UBE3A-mediated ubiquitination, regulating glycolysis and lipid metabolism in the NAFLD process, and contributing to the onset and progression of NAFLD (124). Ubr1 is a RING ubiquitin ligase and a critical amino acid receptor in the hepatocytes. When essential amino acids are depleted, Ubr1 becomes inactive in hepatocytes and fails to catalyze the proteasome-mediated degradation of the lipid droplet protective protein perilipin 2 (Plin2) via polyubiquitination. Elevated Plin2 protein levels inhibit hepatic lipolysis, resulting in NAFLD in lean individuals (125). This study identified a novel mechanism by which NAFLD develops in lean individuals and suggested that hepatic steatosis can be alleviated by activating Ubr1 through dietary essential amino acid supplementation (particularly leucine and isoleucine) to degrade Plin2, indicating a promising strategy for treating NAFLD in lean individuals. S-phase kinase-associated protein 2 (Skp2) is a multi-subunit RING ubiquitin ligase. Overexpression of Kindlin-2 in the liver exacerbates NAFLD by promoting defective lipid metabolism and inflammation in hepatocytes (126). Molecular biology and biochemical research has indicated that the C-terminal region of Kindlin-2 binds to Foxo1 and inhibits Skp2-mediated ubiquitination of the Foxo1 protein, enhancing Foxo1 protein stability and inducing hepatic steatosis (126).

In addition to these enzymes, other E3 ubiquitin ligases and deubiquitinating enzymes have also been implicated in the pathogenesis of NAFLD. For example, mouse double minute 2 homolog (MDM2) is a RING ubiquitin ligase. USP36 is a deubiquitinating enzyme. Enhancer of liver regeneration (ALR) is a sulfhydryl oxidase and cytochrome C reductase that plays a crucial role in mitochondrial dynamics (127). According to reports, MDM2 and USP36 can operate on ALR simultaneously, with MDM2 promoting proteasome-mediated degradation via ubiquitination and USP36 deubiquitinating ALR (128). An imbalance between ubiquitination and deubiquitination of the ALR leads to severe degradation of the ALR, which enhances mitochondrial division and severely impairs oxidative phosphorylation (OXPHOS) and ATP synthesis (128). Abnormal mitochondrial fragmentation due to ALR depletion may be a key aggravating factor in the progression of NASH-HCC and a promising therapeutic target for related liver diseases. Another study found that MDM2 enhances recombinant heat shock transcription factor 1 (HSF1) ubiquitination and inhibits NLRP3, which activates inflammatory vesicles and boosts NAFLD and IR (129). These two studies demonstrate that MDM2 has opposing regulatory roles in NAFLD. RPN11 is a crucial component of the proteasome 19S regulatory particle and possesses deubiquitinating enzyme activity. In yeast, RPN11 is an essential subunit of the 19S regulatory particle of the proteasome (130). Recent studies have examined the involvement of RPN11 in NAFLD. RPN11 knockout mice exhibited no effects on diet-induced hepatic steatosis, IR, or steatohepatitis (131). RPN11 deubiquitinates and stabilizes methyltransferase-like 3 (METTL3), promotes m6A modification and the expression of cyl-CoA synthetase short-chain family member 3 (ACSS3), and upregulates lipid metabolism genes through histone propionylation to create propionyl coenzyme A (131). The RPN11-METTL3-ACSS3-histone propionylation pathway is activated in the livers of patients with NAFLD. Capzimin, a pharmacological inhibitor of RPN11, alleviated NAFLD, NASH, and related metabolic disorders in mice while also lowering the lipid content of human hepatocytes in both 2D and 3D cultures (131). These results suggest that RPN11 may be a novel factor promoting lipid metabolism disorders in NAFLD/NASH and that inhibition of RPN11 has therapeutic potential. USP15 also regulates cellular metabolism and energy homeostasis, and its abnormalities may contribute to the onset of metabolic disorders (132). Researchers discovered that The expression of the deubiquitinating enzyme USP15 is upregulated in the livers of HFD-fed mice and in liver biopsy tissues from patients with NAFLD or NASH, whereas hepatocyte deletion of USP15 suppresses hepatic steatosis, inflammation, and fibrosis in a mouse model of NASH (133). USP15 can deubiquitinate FABPs and Perilipin proteins, thereby increasing their stability and boosting lipid accumulation (133).

## 5 Conclusion and prospects

E3s and DUBs have emerged as key regulators of NAFLD. Despite significant progress in elucidating their functions and mechanisms, among others, the understanding of the roles of E3s and DUBs in NAFLD remains limited. To date, only a few DUBs have been shown to be involved in NAFLD. Therefore, there is an urgent need to search for new DUBs involved in NAFLD. Ferroptosis, intestinal microecological imbalance, and epigenetic inheritance are also important factors affecting the development of NAFLD, and E3s and DUBs may be involved in NAFLD by regulating these processes. Furthermore, one type of E3 or DUB may have many substrates that regulate the pathogenesis of NAFLD, such as TRAF6, TRIM8, MDM2, and Nedd4L. Meanwhile, important proteins involved in the development of NAFLD may be regulated via multiple E3s or DUBs, such as FASN, which can be regulated by USP14, TRIM28, and TRIM56. There are some inconsistent findings regarding the role of various E3s in NAFLD, such as Hrd1, MDM2, and Nedd4L. Research suggests that they may play a role in promoting or preventing the occurrence and progression of NAFLD. Therefore, further studies are needed to clarify their role in NAFLD. Currently, most studies are limited to observing changes in the expression of these enzymes in NAFLD/NASH and lack in-depth exploration of mutations, expression differences, and targeted therapies in patients. For example, TRIM56 has been shown to delay NAFLD progression by ubiquitination modification of the key protein for fatty acid synthesis, FASN, but this study is mainly based on animal models and *in vitro* experiments, and has not yet delved into the existence of relevant mutations or expression differences in patients. Similarly, upregulation of TRIM8 expression in NAFLD is closely associated with disease progression, but its mutation status and targeted therapeutic potential in clinical samples remain unclear. In addition, the protective role of the deubiquitinating enzyme OTUB1 in NASH has been revealed, but its differential expression in patients and its value for clinical applications still need further investigation. No large-scale clinical trials of targeted therapies against E3s or DUBs have been reported in patients with NAFLD/NASH. Currently, TRIM31 and TRIM8 are considered highly promising therapeutic targets, and researchers have embarked on drug-targeting studies against TRIM31 and TRIM8 to explore their application in NAFLD therapy. However, the clinical application of these targets is still in the early exploratory stage and has not yet entered clinical trials.

In summary, although basic research has provided important clues for understanding the roles of E3s and DUBs in NAFLD/NASH, further in-depth studies on the mutations and expression differences of the relevant enzymes as well as the efficacy and safety of targeted therapies in patients are needed to translate these findings into clinical therapeutic strategies, and to provide more than enough research evidence for the translation of clinical drugs.

## Glossary

NAFLD: non-alcoholic fatty liver disease
NASH: non-alcoholic steatohepatitis
HCC: hepatocellular carcinoma
IR: insulin resistance
ERS: endoplasmic reticulum stress
E3s: E3 ubiquitin ligases
DUBs: deubiquitinating enzymes
Ub: Ubiquitin
TAK1: transforming growth factor-beta-activated kinase 1
MAP3K7: mitogen-activated protein kinase kinase 7
JNK: c-Jun N-terminal kinase
NF-κB: nuclear factor kappa-B
Rhbdf2: rhomboid family member 2
TRAF6: TNF receptor associated factor 6
TRAF: TNF receptor associated factor
RF: ring finger domain
ZF: zinc finger motif
IKKβ: inhibitor of nuclear factor kappa-B kinase beta
MKK: MAPK kinase
IRS1: insulin receptor substrate 1
AKT: protein kinase B
GSK3β: glycogen synthase kinase 3 beta
FOXO1: forkhead box protein O1
IRHOM2: rhomboid protein 2
CYLD: Cylindromatosis
ASK1: Apoptosis Signal Regulating Kinase 1
NAFL: non-alcoholic fatty liver
FBXW5: F-box and WD repeat domain containing 5 gene
OTUB1: OTU domain-containing ubiquitin aldehyde-binding proteins Otubain1
HFHC: high-fat high-cholesterol
PIKK: PI3K-associated kinase
mTORC1: mTOR complex 1
mTORC2: mTOR complex 2
SREBP: sterol response element binding protein
FBW7: F-box and WD repeat domain-containing7
ApoJ: Apolipoprotein J
CUL4B-DDB1: Cullin 4B-Ring E3 ubiquitin ligases
PPARα: peroxisome proliferator activated receptor alpha
S6K1: ribosomal protein S6 kinase beta-1
S6K2: ribosomal protein S6 kinase beta-2
BCAAs: branched-chain amino acids
BCKAs: branched chain α-ketoacids
CUL4A: Cullin 4A
SHIP2: src-homology2-containing inositol 5-phosphatase 2
DDX5: DEAD-box protein 5
HFD: high-fat diet
NLRP3: pyrin domain containing protein 3
HK: Hyperforcinol K
CREB: cAMP response element binding
ZNF638: zinc finger protein 638
GRAIL: gene related anergy in lymphocyte
ATGL: adipose triglyceride lipase
TG: triglyceride
FA: fatty acid
TGF-β: transforming growth factor-β
MFB: myofibroblast
ECM: extracellular matrix
TrkB: tyrosine kinase receptor B
ARID2: AT-rich interaction domain 2
CARM1: coactivator associated arginine methyltransferase1
UPR: unfolded protein response
ERAD: ER-associated protein degradation
TXNIP: thioredoxin-interacting protein
TRIB3: tribbles homolog 3
HNF4α: hepatocyte nuclear factor 4 alpha
HFF: high fat diets
LXR: liver X receptor
HSP90AA1: heat shock protein 90alpha family class A member 1
PINK1: parkin and PTEN-induced potential kinase protein 1
Miz1: myc-interacting zinc finger protein 1
PRDX6: peroxiredoxin 6
LDs: lipid droplets
HMGB1: high mobility group protein 1
Hrd1: HMG-CoA reductase degradation protein 1
Acly: ATP citrate lyase
UBE3A: ubiquitin protein ligase E3A
PDHA1: pyruvate dehydrogenase E1 component subunit alpha 1
ACAT1: acetyl-coenzyme A acetyltransferase 1
Plin2: perilipin 2
Skp2: S-phase kinase associated protein 2
MDM2: mouse double minute 2 homolog
ALR: regeneration
HSF1: heat shock transcription factor 1
METTL3: methyltransferase-like 3
CoA: coenzyme A
ACSS3: cyl-CoA synthetase short-chain family member 3
